# Molecular detection and identification of piroplasms in sika deer (*Cervus nippon*) from Jilin Province, China

**DOI:** 10.1186/s13071-016-1435-3

**Published:** 2016-03-16

**Authors:** Junlong Liu, Jifei Yang, Guiquan Guan, Aihong Liu, Bingjie Wang, Jianxun Luo, Hong Yin

**Affiliations:** State Key Laboratory of Veterinary Etiological Biology, Key Laboratory of Veterinary Parasitology of Gansu Province, Lanzhou Veterinary Research Institute, Chinese Academy of Agricultural Science, Xujiaping 1, Lanzhou, Gansu 730046 P. R. China; Jiangsu Co-innovation Center for Prevention and Control of Important Animal Infectious Diseases and Zoonoses, Yangzhou, 225009 P. R. China

**Keywords:** Piroplasmosis, *Theileria*, *Babesia*, Sika deer

## Abstract

**Background:**

Piroplasmosis is an important disease of domestic animals and wildlife and is caused by organisms from the genera *Theileria* and *Babesia*. Wildlife such as sika deer play an important role as reservoir hosts for several species of *Theileria* and *Babesia*. Using blood samples collected from sika deer, we investigated the epidemiology of *Theileria* spp. and *Babesia* spp. in sika deer from Jilin Province in China and identified those species that cause pathogenic infections in sika deer.

**Methods:**

Sixty-eight blood samples of sika deer were collected from three areas of the Jilin Province in Northeast China. Genomic DNA was extracted, and the V4 hypervariable region of the 18S rRNA of the piroplasms was amplified using the nested PCR method. The selected positive samples were sequenced to identify species of *Babesia* and *Theileria*.

**Results:**

PCR detection revealed that 24 samples were positive for *Theileria* and *Babesia* spp. (35.29 %, 95 % CI = 11.8-46.8). After alignment, a sequenced fragment for *Theileria cervi* was found to be the most prevalent from the obtained samples (22.06 %, 95 % CI = 11.8-49.6). Six sika deer samples were identified as being infected with a *Theileria* sp. that was similar to a *Theileria* sp. found from spotted deer in India. In addition to the results above, for the first time, we identified *T. annulata* infection from one sample of sika deer and *Babesia* sp. from two samples, which showed high identity with *Babesia motasi* found in sheep from China.

**Conclusion:**

The present study offers new data on the pathogens of piroplasmosis in sika deer in northeastern China. For the first time, sika deer was confirmed as a reservoir host for the *T. annulata* of cattle and the *B. motasi* of sheep, which was found in China.

## Background

Piroplasmosis is an important tick-borne disease of domestic and wild ruminants and is caused by different species of *Theileira* and *Babesia* all around the world. *Theileria* spp. are cosmopolitan tick-borne protozoan pathogens that can infect both livestock and wild ruminants [1]. Depending on the pathogenicity, *T. annulata* and *T. parva,* transmitted by species of *Hyalomma* and *Rhipicephalus appendiculatus, * were thought to be the most pathogenic species, which cause lymphoproliferative disease with high mortality rates [2], whereas other *Theileira* species are described as benign pathogenic parasites transmitted by *Rhipicephalus* and *Haemaphysalis* ticks to cattle and small ruminants. Many studies have reported that cervids could be infected with many species of *Theileria. Theileria cervi* is a nonpathogenic parasite found in different deer species including white-tail deer, elk, mule deer and pampas deer, which is transmitted by *Amblyomma americanum* in the USA [3, 4]. *Theileria capreoli* Rukhlyadev was first morphologically described from roe deer; similar *Theileria* species, including *Theileria* sp. ZS TO4, have been described in red deer, roe deer and chamois; and *Theileria* sp. 3185/02 has been described in red deer and roe deer based on analyses of 18S rRNA gene [5–7]. In sika deer, at least three *Theileria* spp. that are different from the species identified in cattle, have been reported in Japan and China [3, 8, 9]. In addition, similar species of *Theileria* were also reported in Chinese water deer from North Korea [1]. In reindeer, two *Theileria* genotypes have been found that are similar to a *Theileria* sp. from white-tailed deer but different from *T. cervi* [10]. Thus, cervids could be the host for many species of *Theileria* and may play important roles in their epidemiology in the wild.

Many of the tick species including *Ixodes ricinus*, *I. scapularis*, *Rhipicephalus microplus* and *Haemaphysalis longicornis* were described as the vector that transmitted the pathogens of babesisosis to livestock and wild animals. *Babesia divergens*, an *Ixodes ricinus-*transmitted parasite of cattle and humans, has been reported in several deer species including reindeer, roe deer and red deer and is widely distributed in Europe [7, 11–13]. *Babesia capreoli* is another species that is prevalent in free-living roe deer and sika deer on the European continent [11, 14, 15]. In America, the main pathogen of deer babesiosis is *Babesia odocoilei,* which has been detected in roe deer, reindeer and elk [16, 17]. Some novel *Babesia* spp. have been identified in different deer species; these include *B. venatorum* (formerly identified as *Babesia* sp. EU1) in roe deer and *B. pecorum* sp. in red deer [18, 19]. Additionally, the pathogens of bovine babesiosis, *B. bigemina* and *B. bovis* have been identified in white-tailed deer [18].

The sika deer, which is a first-grade state protected animal in China, numbers approximately 550,000 head and is mainly concentrated in Northeast China [19]. *Theileria cervi* and another *Theileria* sp. were the first pathogens to be reported as causing cervid theileriosis in sika deer in central China [9]. In Northwest China, *Theileria uilenbergi* and *T. capreoli* have been described in sika deer [20]. However, there have been no epidemiological studies on cervid piroplasmosis in northeastern China. In the present study, polymerase chain reaction assay was carried out to amplify the V4 hypervariable region of the 18S rDNA of the piroplasms, and the sequenced fragments were used for phylogenetic analysis to identify species of *Theileria* and *Babesia*.

## Methods

### Sample collection

During June and July 2015, 68 blood samples were randomly collected from domesticated sika deer from four ranches (DF *n* = 13, TH *n* = 15, SY *n* = 9, and ZJ *n* = 31) in Jilin Province, in northeastern China. Blood samples from the jugular veins were collected into tubes containing EDTA and stored at 4 °C until DNA extraction.

### DNA extraction and primer design

DNA was extracted from 300 μl blood using the Genomic DNA purification Kit (Qiagen, Hilden, Germany) according to the manufacturer’s instructions. The DNA concentration was determined with a NanoDrop 2000 spectrophotometer (Nanodrop Technologies, Wilmington, DE, USA). DNA was stored at -70 °C until further analysis.

### PCR amplification of the V4 region of 18S rRNA gene

The V4 hypervariable region of the 18S rRNA gene, as the target sequence, was amplified by using nested PCR. Universal primers that can amplify a wide variety of *Babesia* and *Theileria* spp. were used [21–23]. The first PCR reaction was conducted using primers RLB-F2 (5’-GACACAGGGAGGTAGTGACAAG-3’) and RLB-R2 (5’-CTAAGAATTTCACCTCTGACAGT-3’). The reactions were performed in a final volume of 25 μl containing 12.5 μl Premix Taq DNA polymerase (TakaRa, China), 1.0 μM of each primer and 1 μl of DNA template. The PCR reaction system comprised one step of initial denaturation at 95 °C for 3 min, followed by 35 cycles of denaturation (95 °C for 1 min), primer annealing (52 °C for 50 s) and extension (72 °C for 1.5 min). The final extension was performed with one step at 72 °C for 5 min. The second PCR was carried out with the primer RLB-FINT (5’-GACAAGAAATAACAATACRGGGC-3’) used as the forward primer together with RLB-R2. The reaction mixture was as in the first PCR reaction, except the template was replaced by 1 μl of the first PCR product. The reaction cycling in the second PCR was optimized for one step of initial denaturation at 95 °C for 3 min, followed by 35 cycles of denaturation (95 °C for 30 s), primer annealing (50 °C for 30 s) and extension (72 °C for 30 s), and the final extension was performed at 72 °C for 5 min. PCR products were electrophoresed on a 1.5 % agarose gel containing 10 μl of Goldview (SolarBio, China) in Tris–acetate-EDTA (TAE) buffer at 120 V for 40 min and visualized under UV light.

The positive PCR products were excised from the gel and purified using an Axygen Gel Purification Kit. The DNA fragment was cloned into the pGEM-T Easy vectors (Promega, 2800 Woods Hollow Road Madison, WI 53711-5399, USA). The *Escherichia coli* JM 109 (TaKaRa, China) was transformed and plasmid DNA from the selected clones was identified using PCR with primers as indicated (program and reaction mixtures were the same as those used in the PCR amplification described above) to verify the presence of correct inserts in selected clones before proceeding with the sequencing process in the Big Dye Terminator Mix of TaKaRa Company (China).

### PCR amplification of a long fragment of the 18S rRNA gene

Samples shown to be positive in the nested PCR were selected for amplification of the long fragment of the 18S rRNA gene, with the aim of confirming the species of *Theileria* and *Babesia*. Two published primers, NBab-1f (5’-AAGCCATGCATGTCTAAGTAGAAGCTTTT-3’) and 18SRev-BT (GAATAATTCACCGGATCACTCG), were used to amplify an approximately 1,600-bp fragment [24, 25]. The reaction mixture was performed in a final volume of 50 μl, with 1.0 μM of each primer, 2 μl of DNA template, 5 μl of 10× High Fidelity buffer, 2 μl of 50 mM MgSO_4_, 4 μl dNTP mixture (2.5 mM of each), and 1 unit of Platinum Taq DNA Polymerase High Fidelity (Invitrogen, USA). The cycling conditions included initial denaturation at 95 °C for 3 min, followed by 35 cycles of 94 °C for 30 s, 58 °C for 30 s and 72 °C for 2 min, with one step of final extension at 72 °C for 10 min. The steps for cloning and sequencing were the same as previously described.

### Sequence analysis

The obtained sequences were aligned using the MegAlign component of the DNAStar software program (Version 4.0 DNAStar, Madison, USA). After alignment with the related *Theileria* and *Babesia* spp. 18S rDNA sequences retrieved from the GenBank, parts of the cloning vector region were removed manually. The resulting sequences were then submitted to the GenBank database.

A phylogenetic tree was generated based on the cloned sequences and the related *Theileria/Babesia* 18S rDNA sequences in GenBank by using the neighbor-joining algorithm in the MEGA 6.0 software [26]. The evolutionary distances were computed using the Kimura two-parameter method [27].

### Statistical analysis

The 95 % confidence intervals (95 % CIs) for the overall prevalence values of *Theileria/Babesia* were calculated using IBM SPSS Statistics version 19.0.

### Ethical approval

The present works were approved by the Animal Ethics Committee of Lanzhou Veterinary Research Institute, CAAS (No. LVRIAEC2013-010). The procedures for acquiring the field samples were approved by the Animal Ethics Procedures and Guidelines of China.

## Results

The DNA of the *Babesia* and *Theileria* spp. samples was detected with nested PCR targeting the V4 region of the 18S rRNA gene. Of the 68 sika deer blood samples, 24 samples were positive for piroplasms (35.29 %, 95 % CI = 11.8-46.8). The PCR products of all 24 positive samples were sequenced and deposited in the GenBank database (accession numbers KT683524-KT683536,KT959214-KT959221, KT970057 and KT970058). After alignment with the related sequences, *T. cervi* was the most prevalent parasite detected from the sika deer. For the sika deer, 15 out of 68 samples (22.06 %, 95 % CI = 11.8-49.6) were positive for *T. cervi*. The second most prevalent parasite was a *Theileria* spp. which had 100 % identity with India spotted deer isolate *Theileria.* sp (JX112750); 6 samples were positive for this *Theileria*. For the first time, *Theileria annulata* was detected in one sika deer (ZJ07) and showed 100 % identity with *T. annulata* (EU083801). In addition to the *Theileria* spp. a *Babesia* sp. was detected in samples DF02 and DF07 and showed 100 % identity with *Babesia* sp. China-BQ1 (AY260181) from sheep.

The aim of this study was to confirm the parasites identified based on the V4 region of the 18S rRNA gene. A fragment of approximately 1,600 base pairs of the 18S rDNA was amplified and sequenced from selected positive samples, especially the samples positive for *T. annulata*, *Babesia* sp. and *Theileria* sp. The obtained sequences were deposited in GenBank (accession numbers KT959223-KT959232). Clustal W from DNAstar software analysis indicated that two types of *Theileria* were found. One type contains 5 clones that have at least 99.3 % identity with *T. cervi* from China and with *Theileria* sp. (CNY3C, yamaguchi and lwate) from Japan. The other type is similar with *Theileria* sp. isolated from India spotted deer, having 99.8 % identity with it. Analysis of the long 18S rDNA sequence showed that the sequence of ZJ07 shared 99.4 % identity with *T. annulata* (EU083801). The sequence of DF02 has 99.3 % identity with *Babesia* sp. China-BQ1 (AY260181) from sheep. Attempts to amplify the long fragment of the 18S rRNA gene from DF07 failed.

**Fig. 1:**
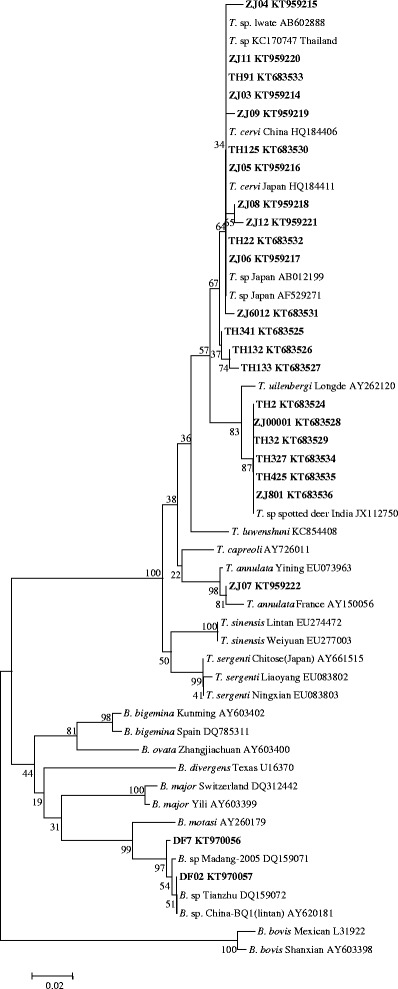
Phylogenetic tree of *Theileria* and *Babesia* spp. based on the V4 region of 18S rRNA gene sequences. The parasite identified in the present study is marked in bold

**Fig. 2:**
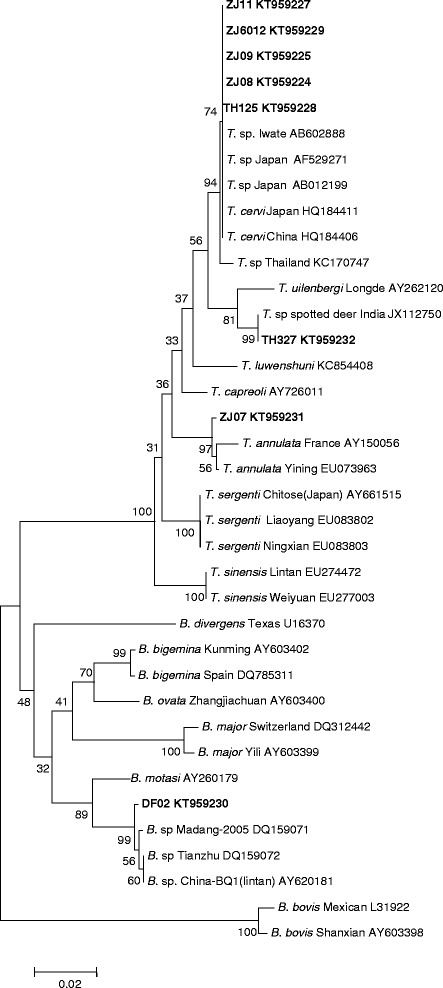
Phylogenetic tree of *Theileria* and *Babesia* spp. constructed based on the 18S rRNA gene sequences. The parasite identified in the present study is marked in bold

The obtained V4 sequences and long 18S rDNA sequences were subjected to phylogenetic analysis along with the related sequences from GenBank with the MEGA 6.0 software. The two phylogenetic trees yielded were identical. As shown in Figs. 1 and 2, most of the samples fell into the *T. cervi* clade. However, some samples were different from *T. cervi* and fell into the *Theileria* sp. from the India spotted deer group. The sequence from sample ZJ07 was grouped with *T. annulata*, and DF02 was in the clade of the *B.* sp from sheep.

## Discussion

In the present study, four different sika deer pastures which are close to some small villages containing free-grazing cattle and sheep, were selected for collection of blood samples. During sample collection, some sika deer were found losing weight and suffering anemia, in addition with the detection of *T. annulata* and *T. sergenti* from cattle from the same area [28]. It is possible for sika deer infected with piroplasms. Among wildlife, species of the Cervidae are not only the hosts for piroplasms that affect different species of deer, but could also be the hosts for many bovine piroplasms. The pathogens of bovine babesiosis, including *B. bigemina* and *B. bovis*, have been reported in infected white-tailed deer, pampas deer, brown brocket deer and marsh deer from the United States and Brazil [4, 18, 29]. Another bovine *Babesia* spp. found from deer is *B. divergens,* which is also a main pathogen of human babesiosis in Europe [30, 31]; many deer species, including reindeer, roe deer and red deer, have been described as infected with this parasite [11, 32, 33]. To date, no report exists of the agent of bovine theileriosis being found in cervids. Only a single *Theileria* spp. identified from sika deer has shown 97 % identity with *T. sergenti,* but the species was distinct from the clade of cattle *Theileria* spp. in the phylogenetic tree based on an analysis of 18S rRNA genes [3].

Sixty-eight DNA samples of sika deer were first evaluated with a nested PCR method for the detection of piroplasms [21–23]. From the PCR results, 24 samples were found to be positive for *Theileria* or *Babesia*. Because an aim of the present study was to clarify the genus of the parasites found, the V4 sequence of all positive samples was sequenced. Sequence analysis allowed the identification of two types of *Theileria,* which were the same *T. cervi* and *Theileria* sp. found in Indian spotted deer. Because *T. cervi* can infect different deer species and has been found previously in sika deer in China [3, 9, 34], the finding of *T. cervi* in sika deer in the present study was not surprising. Of more interest was the identification of *T. annulata* in sika deer based on the analysis of 18S rDNA sequences. In the phylogenetic tree constructed based on the 18S rDNA sequences, the *T. annulata* isolated from the sika deer fell into the clade including the *T. annulata* isolates from China and other countries. The percent identity of *T. annulata* isolated from sika deer with the *T. annulata* Yining (EU083801) isolate was 99.4 %. To the best of our knowledge, *T. annulata* had only been isolated previously from infected sheep and bovidae. No other information on the organisms that cause bovine theileriosis has been identified from other ruminants. This is the first identification of *T. annulata* in sika deer and extends the host range for this parasite.

As previous studies have described, the pathogens of cervine babesiosis include *B. capreoli*, *B. venatorum (*formerly as *Babesia* sp. EU1), *B. divergens* and *B. pecorum* [35]. In the present study, none of the *Babesia* spp. described above were found, but *B. motasi* infection was identified in two sika deer. *B. motasi* is one of the most important pathogens of ovine babesiosis in China. At least 6 isolates of *B. motasi* have been collected by our institute [36]. The *Babesia* parasite found in the present study showed 100 % identity on the V4 region and 99.3 % identity with the long fragment of the 18S rDNA with *Babesia* sp. China-BQ1 (Lintan), which indicated the parasite is *B. motasi*. Previous studies have reported that *B. motasi* can infect wild Caprinae, such as the Alpine ibex and chamois but no other animals [13]. Our finding is the first to describe sika deer as the host for *B. motasi*.

From Jilin province, many tick species have been reported including *Hyalomma scupense*, *Hy. detritum* Schulze which are the vectors for *T. annulata,* and *Hae. longicornis* for the transmission of *B. motasi* [37]; the vector for *T. cervi* in China is still unclear. In Japan, *Hae. longicornis*, *Hae. flava*, *Hae. yeni*, *Hae. megaspinosa*, *I. ovatus* and *Amblyomma testudinarium* were detected on sika deer, but the piroplasms were not determined from the ticks [3, 38].

As described before, livestock could introduce disease to wildlife, which can act as a maintenance host and, therefore, as the source of infection for livestock [39]. Therefore, in future studies, confirmation of the ability of the *T. annulata* and *B. motasi* that were isolated from sika deer to infect their original hosts is needed, thus helping with the control of the disease in both livestock and wildlife.
