# Erratum to: Molecular detection and identification of piroplasms in sika deer (Cervus nippon) from Jilin Province, China

**DOI:** 10.1186/s13071-016-1475-8

**Published:** 2016-04-01

**Authors:** Junlong Liu, Jifei Yang, Guiquan Guan, Aihong Liu, Bingjie Wang, Jianxun Luo, Hong Yin

**Affiliations:** State Key Laboratory of Veterinary Etiological Biology, Key Laboratory of Veterinary Parasitology of Gansu Province, Lanzhou Veterinary Research Institute, Chinese Academy of Agricultural Science, Xujiaping 1, Lanzhou, Gansu 730046 P. R. China; Jiangsu Co-innovation Center for Prevention and Control of Important Animal Infectious Diseases and Zoonoses, Yangzhou, 225009 P. R. China

## Erratum

Unfortunately, the original version of this article [1] contained an error. Within the results section, and in Fig. 1, the accession numbers KT683524-KT683536 should be KT863524-KT863536. The correct version of Fig. 1 can be found below.

**Fig. 1:**
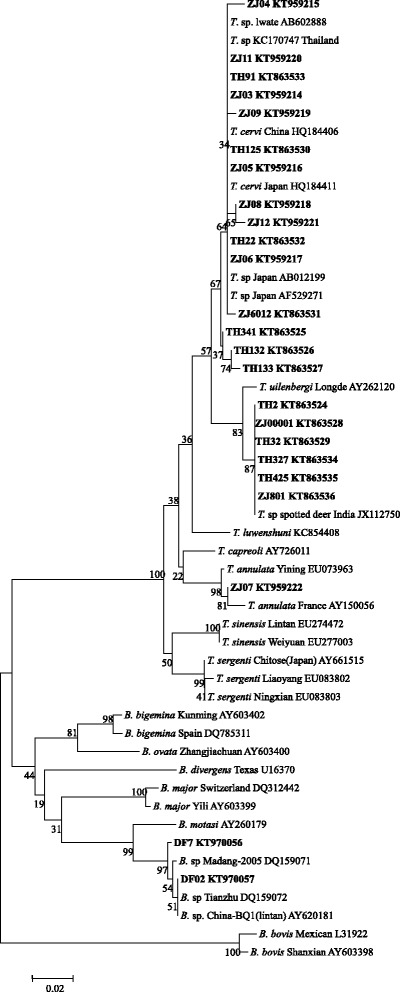
Phylogenetic tree of Theileria and Babesia spp. based on the V4 region of 18S rRNA gene sequences. The parasite identified in the present study is marked in bold

We would like to apologize for this error and for any inconvenience this may have caused.
